# The value of kidney injury molecule 1 in predicting acute kidney injury in adult patients: a systematic review and Bayesian meta-analysis

**DOI:** 10.1186/s12967-021-02776-8

**Published:** 2021-03-12

**Authors:** Jiwen Geng, Yuxuan Qiu, Zheng Qin, Baihai Su

**Affiliations:** 1 Department of Nephrology, West China Hospital, Sichuan University, Chengdu, 610041 China; 2 Department of Ultrasound, West China Hospital, Sichuan University, Chengdu, 610041 China

**Keywords:** Kidney injury molecule 1, KIM-1, Acute kidney injury, AKI, Meta-analysis, Systematic review

## Abstract

**Introduction:**

The aim of the study was to systematically review relevant studies to evaluate the diagnostic value of urinary kidney injury molecule 1 (uKIM-1) for acute kidney injury (AKI) in adults.

**Method:**

We searched PubMed and Embase for literature published up to November 1st, 2019 and used the Quality Assessment Tool for Diagnosis Accuracy Studies (QUADAS-2) to assess the quality. Then, we extracted useful information from each eligible study and pooled sensitivity, specificity, and area under the curve (AUC) values.

**Results:**

A total of 14 studies with 3300 patients were included. The estimated sensitivity of urinary KIM-1 (uKIM-1) in the diagnosis of AKI was 0.74 (95% CrI 0.62–0.84), and the specificity was 0.84 (95% CrI, 0.76–0.90). The pooled diagnostic odds ratio (DOR) was 15.22 (95% CrI, 6.74–42.20), the RD was 0.55 (95% CrI 0.43–0.70), and the AUC of uKIM-1 in diagnosing AKI was 0.62 (95% CrI 0.41–0.76). The results of the subgroup analysis showed the influence of different factors.

**Conclusion:**

Urinary KIM-1 is a good predictor for AKI in adult patients with relatively high sensitivity and specificity. However, further research and clinical trials are still needed to confirm whether and how uKIM-1 can be commonly used in clinical diagnosis.

## Introduction

Acute kidney injury (AKI) is part of a series of acute kidney diseases during the renal disease processes [1]. Currently, it is determined by an abrupt increase in serum creatinine, a decrease in glomerular filtration rate, or both [2]. AKI is a common and serious disease among patients with acute illness in nearly all fields of medical practice [3]. It is a global issue and can be fatal if not treated well [4]. A recent epidemiologic study demonstrated that the incidence AKI was 21.6% and that the AKI-associated mortality rate was 23.9% in adults [5]. The early diagnosis and intervention of AKI can not only provide better opportunities for alternative therapeutic options but also improve patient prognosis and reduce mortality. Although an increasing number of studies have deepened our understanding of AKI, the clinical diagnostic criteria of AKI remain controversial. At present, AKI is usually diagnosed by an increased serum creatinine or decreased urine output, which was supported by the RIFLE (risk, injury, failure, loss, end stage kidney disease) classification in 2004 [2], the AKIN (acute kidney injury network) classification in 2007 [6], and the KDIGO (Kidney Disease Improving Global Guidelines) in 2012 [7]. However, both serum creatinine and urine output have limitations in timely and accurately recognizing decreased renal function, resulting in poor sensitivity and specificity in diagnosing AKI [8]. All these findings suggest an urgent need for more effective diagnostic measurements for AKI, and an increasing number of scholars have started related research in an attempt to change this situation.

Over the last few years, several novel AKI biomarkers have been discovered and characterized. Some of them are considered to have the potential to help diagnose AKI early, including neutrophil gelatinase-associated lipocalin (NGAL), interleukin-18 (IL-18), kidney injury molecule-1 (KIM-1), and tissue inhibitor of metalloproteinase 2 (TIMP-2) and so on [3, 9]. Unfortunately, none of them has sufficient evidence to replace serum creatinine as a marker for measuring renal function or as a diagnostic criterion for AKI. Among these sundry kinds of new biomarkers, many scholars have demonstrated that urinary KIM-1 (uKIM-1) is an remarkably predictive marker for AKI detection. Moreover, KIM-1 has been approved by the US Food and Drug Administration (FDA) as an AKI biomarker for preclinical drug development [10]. Its expression is upregulated in the early stages of AKI, much earlier than when serum creatinine increases, providing more possibilities for treating AKI [3].

KIM-1 is a 38.7-kDa type I transmembrane glycoprotein with an extracellular immunoglobulin-like domain topping a long mucin-like domain. As usual, it is expressed at low levels in the kidney and other organs, but it is significantly upregulated when the kidney undergoes injury, especially after ischemia–reperfusion injury [11]. In humans, proximal tubule cells are the main locations where KIM-1 expression is upregulated [12]. KIM-1 plays an important role in both kidney injury and the associated recovery processes [13]. Hence, some studies have proposed uKIM-1 as a sensitive and specific marker of AKI as well as a predictor of outcome [14]. Although an increasing number of related studies have been conducted in recent years, additional clinical research and trials are required to support the clinical application of KIM-1 in the early diagnosis of AKI [15].

To further evaluate the diagnostic value of uKIM-1 for AKI, we systematically reviewed relevant studies to further clarify the predictive performance and diagnostic value of uKIM-1 in AKI. Since previous research suggested that age might be a critical factor affecting the diagnostic value of uKIM-1, we included 14 original articles on studies conducted only on adults. We selected the Bayesian bivariate model as our main analytical method, as compared with other common methods, it has higher accuracy and is not affected by heterogeneity [16].

## Methods

This meta-analysis was carried out according to the preferred reporting items for systematic reviews and meta-analyses (PRISMA) statement [17].

### Data sources and search strategy

A comprehensive search of literature published up to November 1st, 2019, was performed in the following databases: PubMed (Medline) and Embase. The search strategy was applied to identify all trials with the following keywords: kidney injury molecule 1 or KIM-1 plus acute kidney injury or acute renal failure. In addition, the reference lists of all included studies and relevant reviews were scanned. The searches were performed independently by 2 researchers (J Geng and Z Qin).

### Study selection

We encompassed all articles and conference papers retrieved without sample size restrictions. Studies that complied with the following criteria were finally retrieved: (1) articles and conference papers that had a prospective cohort design, a case–control design or a cross-sectional design and explored the performance of urinary KIM-1 in the detection of AKI; (2) study subjects all older than 18 years; and (3) studies that included or allowed calculation of the estimated sensitivity and specificity of urinary KIM-1 in the diagnosis of AKI. The two reviewers (J Geng and Z Qin) used the EndNote bibliography manager to check the titles and abstracts of all citations and then retrieved and rescreened the full-text articles. The reference lists of the reviewed full-text articles were checked to ensure no additional relevant studies were missed. Any discrepancies were resolved by a third researcher (Y Qiu).

### Data extraction

One reviewer (J Geng) utilized a standardized form to extract information from each eligible study. The following information was extracted: (1) research information: first author, year of publication, country of origin, study design, sample size, whether the investigators were blinded, population setting (patients after cardiopulmonary bypass surgery, patients with coronary angiography or percutaneous coronary interventions, patients with solid tumors, patients with malaria, patients in general hospital ward, patients admitted to the intensive care unit, patients admitted to the emergency department, and critically ill patients); (2) Characteristics of the study subjects: age, sex, basal estimated glomerular filtration rate, and baseline serum creatinine; (3) AKI information: definition of AKI and number of AKI patients; (4) KIM-1 information: timing of measurements, measurement method, and the value of KIM-1; and (5) information about the outcomes, such as the optimal cutoff thresholds, the sensitivity and specificity and/or the true positive, true negative, false positive, and false negative values. If a study proposed more than one cutoff threshold, we used the cutoff value with the highest product of specificity and sensitivity.

### Evidence quality assessment

The methodological quality of the studies was individually evaluated by one author (J Geng). We used the Quality Assessment Tool for Diagnosis Accuracy Studies (QUADAS-2) [18] to evaluate the quality of each trial. The tool included 4 domains: patient selection, index test, reference test, and flow and timing. We judged a study as having a low risk of bias if it was evaluated as low on all 4 domains or the first 3 terms concerning applicability. Otherwise, we judged it as having a high risk of bias. Any discrepancies were resolved by a third researcher (Y Qiu).

### Data synthesis and analysis

A Bayesian bivariate model for diagnostic test studies was implemented. The greatest advantage of this method is that adding a small amount of information can stabilize the analysis without overpowering the existing data. Especially when there are few data available, the prior for the covariance matrix of the bivariate structure is important. This provides a basis for the method to provide higher accuracy. Accurate posterior marginal distributions for sensitivity and specificity as well as all hyperparameters and covariates are directly obtained by the bivariate model with no need for Markov chain Monte Carlo (MCMC) sampling [16]. Furthermore, univariable estimates of sensitivity and specificity with 95% credible intervals (CrIs), as well as the summary receiver operating characteristic (SROC) curves, are directly available for interpretation. Moreover, area under the receiver operating characteristic curve (AUC) values with 95% CrI were calculated. Summary positive and negative likelihood ratios (PLRs and NLRs, respectively) were calculated from the summary sensitivity and specificity estimates. Four models were applied, where model type = 1 indicates that the sensitivity (se) and specificity (sp) are modeled in the bivariate model, and model types = 2, 3 and 4 indicate that the sensitivity (se) and false negative rate(1-sp), false positive rate (1-se) and specificity (sp), and false positive rate (1-se) and false negative rate (1-sp) are modeled in the bivariate model, respectively. Model selection was performed according to the deviance information criterion (DIC). Alternative models were compared by using the DIC and considering a difference in DIC score > 5 as important. Funnel plot asymmetry was further examined to allow a valid assessment of the extent and impact of publication and selective reporting bias in studies of diagnostic accuracy. Subgroup analyses were performed according to study design (prospective or retrospective), whether blinding was or was not performed, population settings and assay method. All analyses were conducted by R software 3.6.2 (R Foundation for Statistical Computing, Vienna, Austria; https://www.r-project.org) and RStudio 1.2.5033 (RStudio, Inc., Boston, MA, USA) software with the R packages meta4diag 2.0.8 and INLA 19.09.03 and relied packages.

## Results

### Search results

**Fig. 1:**
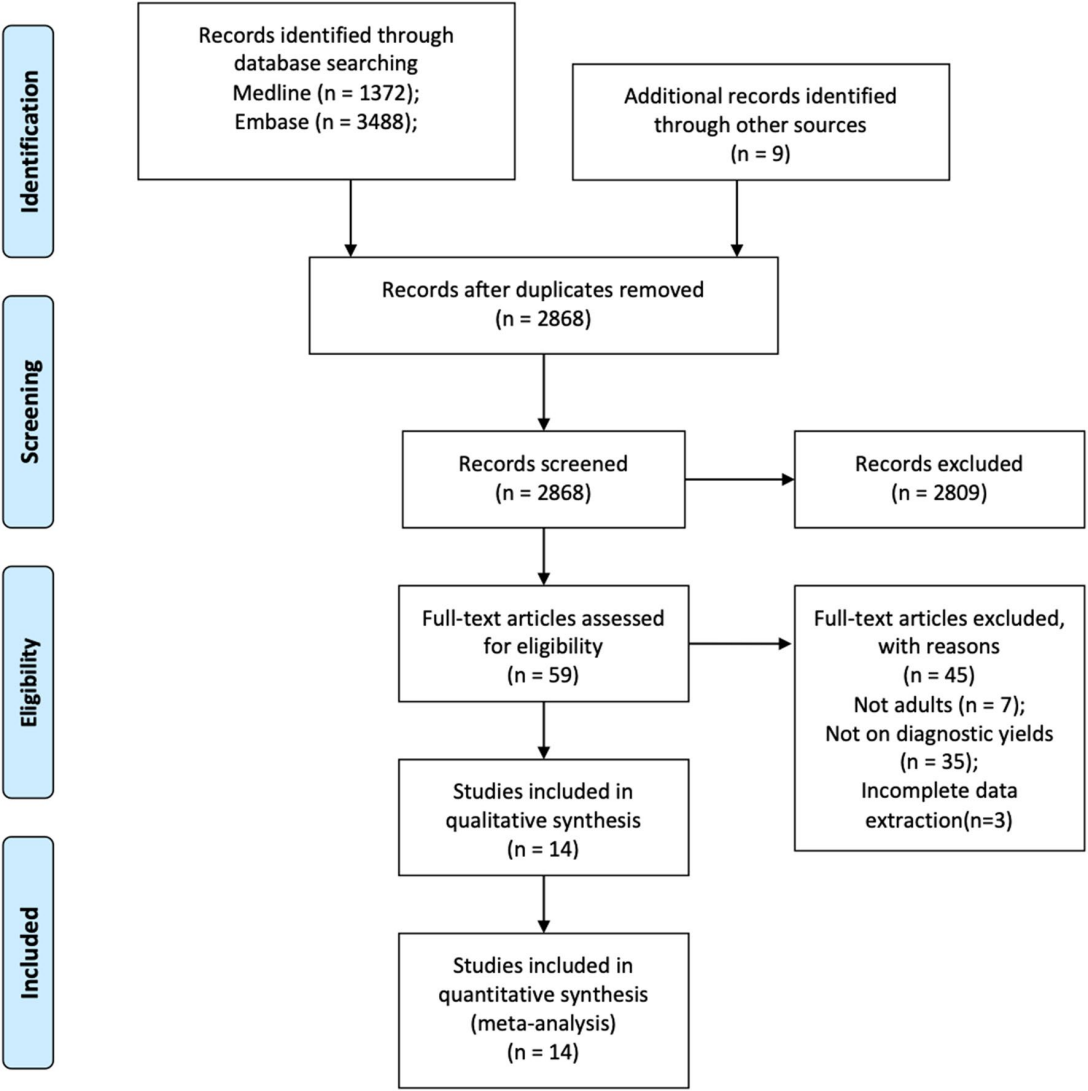
Flow chart of the study identification and selection procedures

The initial search identified 4869 records in total. First, 2001 studies were removed after duplicates were identified. Then, we screened the titles and abstracts of the remaining 2868 studies. There were 59 studies selected for full-text review. Finally, 14 studies were included in this meta-analysis [19–32] (Fig. 1).

### Study and patient characteristics

A total of 14 studies with 3300 patients were included. The characteristics of the individual studies are listed in Table 1. The research objects of all articles were adults (aged above 18 years old). All these studies were published from 2008 to 2019, with different countries of origin, study designs, sample sizes (from 22 to 1635) and population settings. It is worth noting that these articles have different definitions of AKI. There were 9 prospective cohort studies, 3 case–control studies and 2 cross-sectional studies included in this meta-analysis. In five of the studies, the investigators were blinded, the remaining 9 articles did not mention blinding information. Four studies focused on patients after cardiopulmonary bypass surgery, 2 studies focused on patients in the intensive care unit, 3 studies focused on patients in the general hospital ward, and the remaining studies focused on patients with coronary angiography or percutaneous coronary interventions, patients with solid tumors, patients with malaria, patients admitted to the emergency department, and critically ill patients. Urinary KIM-1 levels were measured by a commercial enzyme-linked immune sorbent assay (ELISA) in 12 studies, and the remaining 2 studies used chemiluminescent microparticle immunoassay or microsphere-based Luminex xMAP technology.

**Table 1:** Characteristics of studies included in the meta-analysis

Study_year	Country	Design	Population settings	Blinding of investigators	Definition of AKI	Sample size	Patients with AKI	Age (y)	Males	Baseline eGRF (ml/min/1.73 m^2^)	Baseline Scr (mg/dl)
Khreba_2019	Egypt	Prospective cohort study	CPB surgery	NR	KDIGO criteria	45	27	46.26	23 (51.1%)	NR	NR
Wybraniec_2017	Poland	Prospective observational study	Patients with coronary angiography/percutaneous coronary interventions	NR	≥ 50% relative or ≥ 0.3 mg/dl absolute increase of SCr at 48 h after procedure	95	9	65	66 (69.5%)	NR	NR
Sinkala_2016	Zambia	Case–control study	General hospital ward	NR	NR	80	13	35.6	50 (62.5%)	NR	6.90^a^
van Wolfswinkel_2016	The Netherlands	Prospective study	Patients with malaria	NR	KDIGO criteria	39	6	45.5^a^	33 (84.6%)	NR	1.26^a^
Torregrosa_2014	Spain	Prospective study	Patients in ICU	NR	RIFLE criteria	193	35	64.6^a^	147 (76.2%)	77.70^a^	1.00^a^
Tekce_2014	Turkey	Prospective cohort study	Patients with solid tumours	NR	AKIN criteria	22	8	57.3^a^	16 (72.7%)	102.91^a^	0.92^a^
Nickolas_2012	USA	Prospective cohort study	Patients in emergency department	YES	≥ 50% increase in SCr more than 3 days and patients exposed to stimuli	1635	96	64.4	855 (52.3%)	70.5	0.9
Naggar_2012	Egype	Case–control study	Critically ill patients	NR	RIFLE criteria	40	20	51.8^a^	16 (40.0%)	83.23^a^	0.90^a^
Endre_2011	New Zealand; Australia; USA	Prospective observational study	Patients in ICU	YES	≥ 0.3 mg/dl or ≥ 50% increase in plasma creatinine from baseline	528	147	60	318 (60.2%)	NR	NR
Liang_2010	China	Case–control study	CPB surgery	YES	RIFLE criteria	122	30	30a	NR	NR	1.01^a^
Ferguson_2010	USA	Cross-sectional study	General hospital ward	NR	≥ 50% increase in SCr	134	92	62.6^a^	81 (60.4%)	NR	NR
Liangos_2009	USA	Prospective cohort study	CPB surgery	YES	Increase in serum creatinine by ≥ 50% in the first 72 h following termination of CPB	103	13	68	74 (71.8%)	73	1.1
Han_2009	USA	Prospective cohort study	CPB surgery	YES	Increase in Scr of ≥ 0.3 mg/dl or 2- to threefold from baseline within the first 72 h after surgery	90	36	63.6^a^	61 (67.8%)	NR	1.04^a^
Vaidya_2008	USA	Cross-sectional study	General hospital ward	NR	RIFLE criteria	204	102	56.9^a^	102 (50%)	NR	NR

*AKI* acute kidney injury, *CPB* cardiopulmonary bypass, *SCr* serum creatinine, *ICU* intensive care unit, *eGRF* estimated glomerular filtration rate, *NR* not reported, *KIDGO* Kidney Disease Improving Global Guidelines, *AKIN* acute kidney injury network, *RIFLE*, risk, injury, failure, loss, end-stage renal disease, *USA* United States of America

^a^Mean baseline SCr level (mg/dl), eGRF (ml/min/1.73 m^2^) or age (y, year)

### Quality assessment

The methodological quality of the studies according to the QUADAS is summarized in Table 2. In the patient selection domain, 3 studies were considered to be at high risk due to their case–control design. All of the other domains were considered to be at low or unclear risk.

**Table 2:** Quality assessment of the included studies

Study_year	Risk of bias				Applicability concerns		
	patient selection	Index test	Reference standard	Flow and timing	patient selection	Index test	Reference standard
Khreba_2019	Low	Unclear	Low	Low	Low	Low	Low
Wybraniec_2017	Low	Unclear	Unclear	Low	Low	Unclear	Unclear
Sinkala_2016	High	Unclear	Unclear	Unclear	High	Low	Unclear
van Wolfswinkel_2016	Low	Unclear	Low	Low	Unclear	Low	Low
Torregrosa_2014	Low	Unclear	Low	Low	Unclear	Low	Low
Tekce_2014	Low	Unclear	Low	Low	Low	Unclear	Low
Nickolas_2012	Low	Low	Unclear	Low	Low	Low	Unclear
Naggar_2012	High	Unclear	Low	Low	Low	Low	Low
Endre_2011	Low	Low	Low	Unclear	Low	Low	Low
Liang_2010	High	Low	Low	Low	Low	Low	Low
Ferguson_2010	Unclear	Unclear	Unclear	Unclear	Low	Low	Unclear
Liangos_2009	Low	Low	Unclear	Low	Low	Low	Unclear
Han_2009	Low	Low	Unclear	Low	Low	Low	Unclear
Vaidya_2008	Unclear	Unclear	Low	Low	Low	Low	Low

The table summarizes the risk of bias and applicability concerns

### Data synthesis

**Fig. 2:**
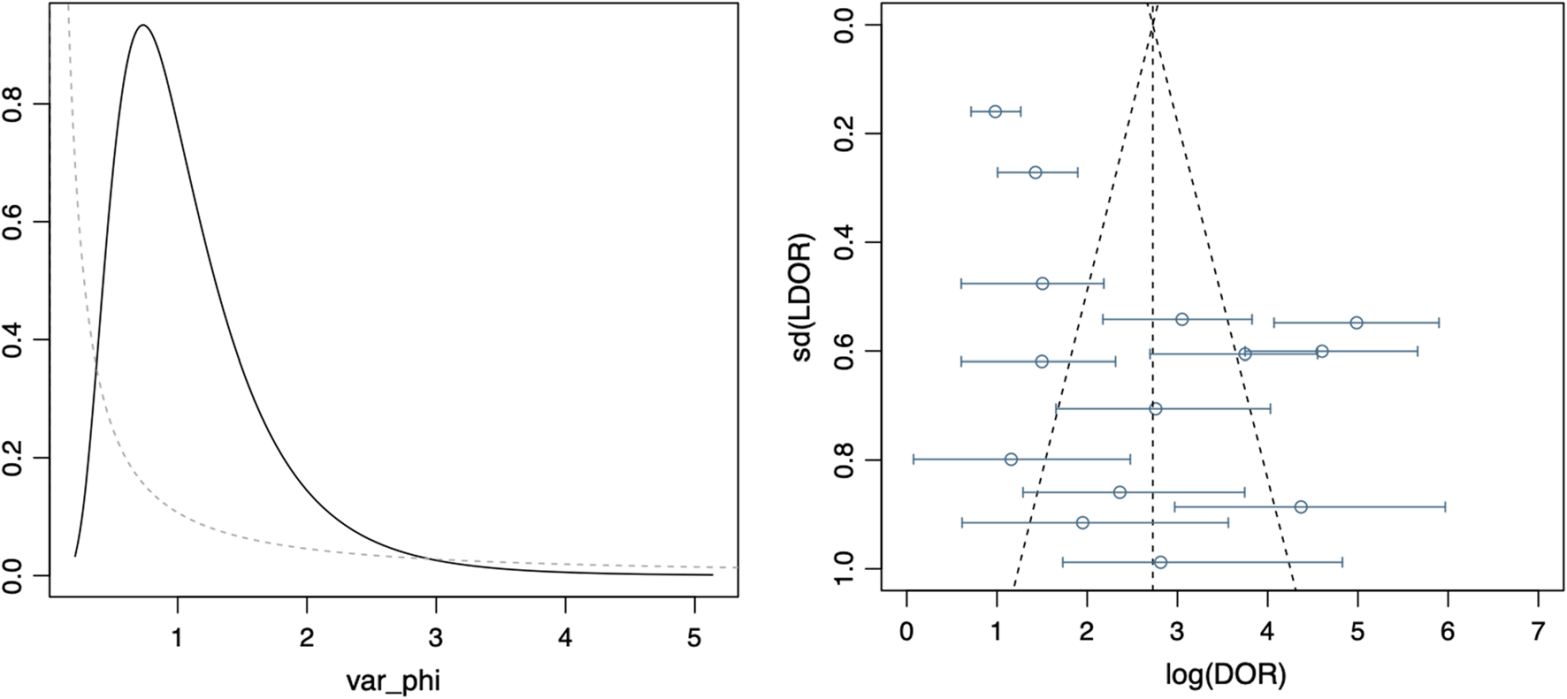
Posterior density distribution plot (left) and Funnel plot (right) for the evaluation of potential bias in diagnosing acute kidney injury with the level of urine kidney injury molecule 1. The posterior density distribution plot displays the consistency of outcomes; the closer the peak is to the coordinate (1, 1), the more consistent the outcomes are (left). In the funnel plot the X-axis represents the diagnostic odds ratio and the Y-axis shows the index of precision of the diagnostic odds ratio (right)

**Fig. 3:**
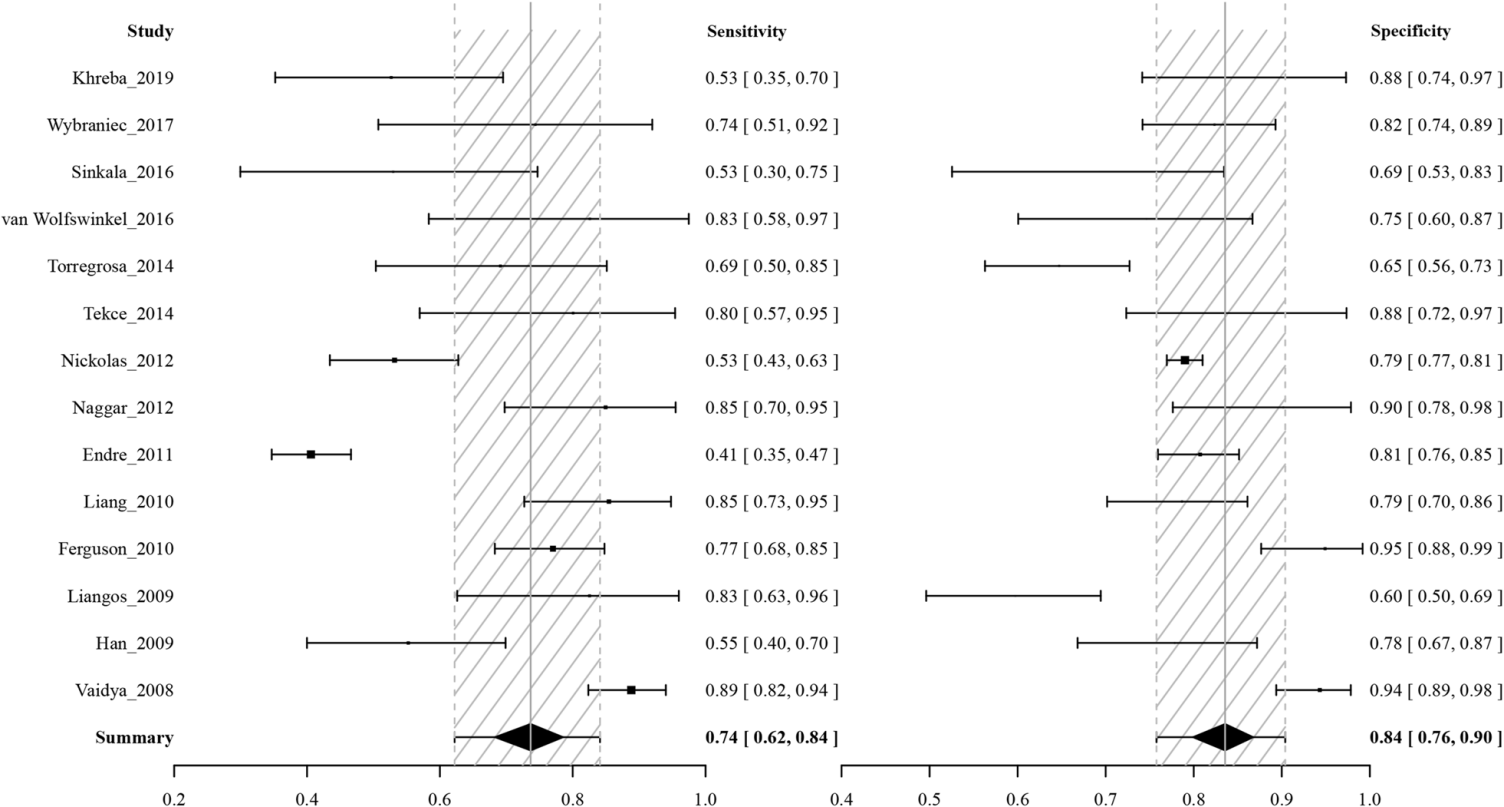
Forest plots of the pooled sensitivity (left), and specificity (right) of the level of urine kidney injury molecule 1 in diagnosing acute kidney injury across all settings. The estimated accuracy for each study is plotted as a point and the 95% credible interval (CrI) is plotted as arrows. A diamond indicates the overall summary point. The gray shaded area represents the 95% CrI of the pooled estimate

**Fig. 4:**
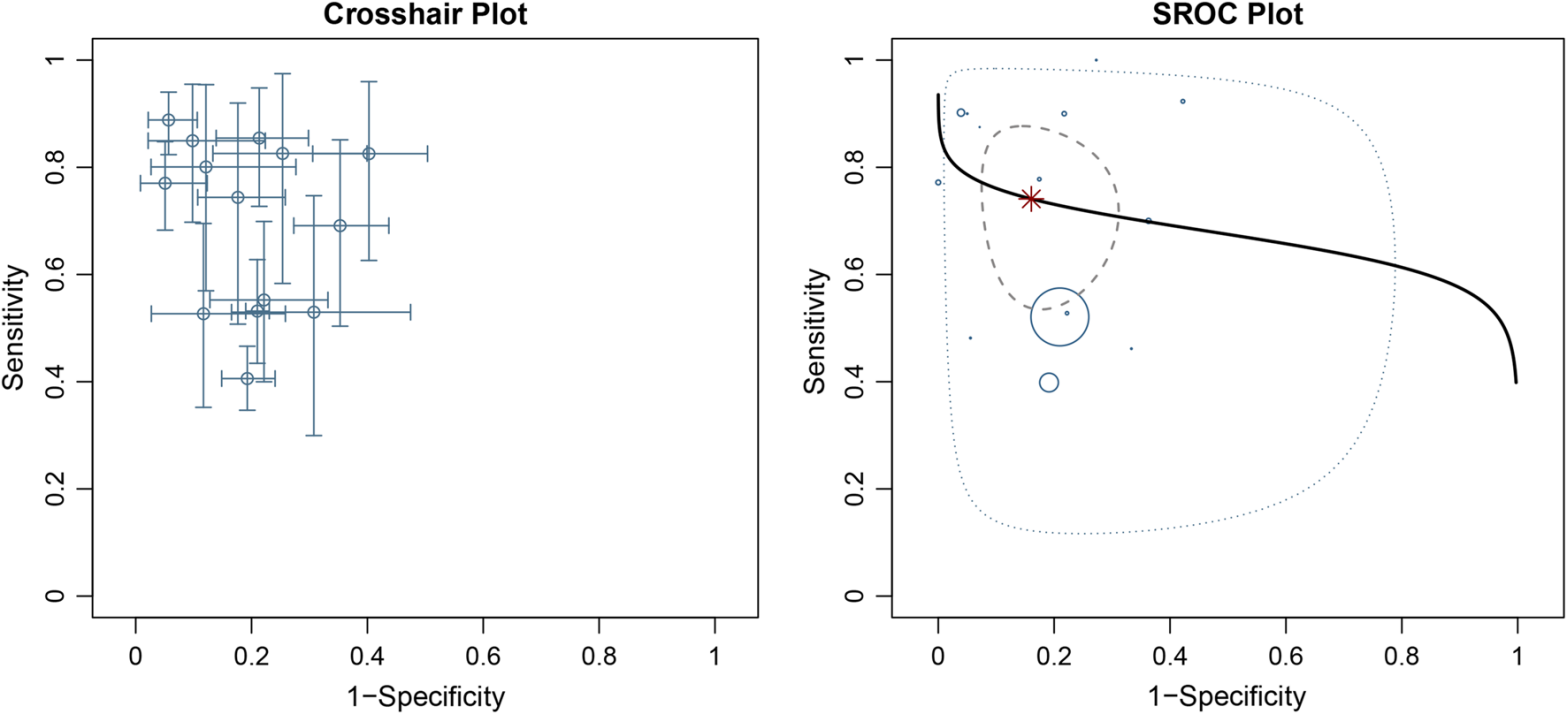
Crosshair plots of the pooled sensitivity (left) and summary receiver operating characteristic (SROC) plots (right) of the level of urine kidney injury molecule 1 in diagnosing acute kidney injury across all settings. The estimated accuracy for each study is plotted as a circle, and the 95% credible interval (CrI) is plotted as arrows (left). The summary receiver operating characteristic line is plotted as a black solid line; the summary point is marked in red; each analyzed study is represented by a circle; the area enclosed by the inner and outer ellipses represents the confidence region and prediction region of the summary points (right)

Data in the 14 eligible studies were extracted and are showed in Table 3, including true positive, false negative, false positive, and true negative values; assay method and time of KIM-1 measurement; and the optimal cutoff values for urinary KIM-1 with their sensitivities (95% CrI), specificities (95% CrI) and AUC-ROC (95% CrI). Our research was based on a Bayesian bivariate model, which was stable and of good consistency, as shown in Fig. 2. Four bivariate models with random effects were analyzed, and no significant difference in the DIC was found (model type = 1, 2, 3, 4: 153.7636, 153.7608, 153.7608 and 153.7636). However, the funnel plots showed that there was significant publication bias (Fig. 2). The estimated sensitivity of urinary KIM-1 in the diagnosis of AKI was 0.74 (95% CrI, 0.62–0.84), and the specificity was 0.84 (95% CrI, 0.76–0.90), as shown in Fig. 3. The pooled diagnostic odds ratio (DOR) was 15.22(95% CrI, 6.74–42.20), and the risk difference (RD) was 0.55 (95% CrI, 0.43–0.70). Crosshair plots showed sensitivity, false positive rate values, and confidence intervals for each included study. The summary receiver operating characteristic (SROC) plot suggested that the efficiency of urinary KIM-1 in AKI diagnosis was considerable (Fig. 4), while the AUC of urinary KIM-1 was 0.62 (95% CrI, 0.41–0.76).

**Table 3:** Performance of urinary KIM-1 for AKI diagnosis in studies included in the meta-analysis

Study_year	TP	FN	FP	TN	Assay method	Timing of measurement	KIM-1 cutoff	Sensitivity (95% CI)	Specificity (95% CI)	AUC (95% CI)
Khreba_2019	13	14	1	17	ELISA	3 h after operation	1.9 ng/mg	0.48 (NR)	0.94 (NR)	0.715 (NR)
Wybraniec_2017	7	2	15	71	ELISA	6 h after CA	0.43 ng/mg	0.778 (NR)	0.824 (NR)	0.81 (NR)
Sinkala_2016	6	7	9	18	ELISA	NR	3.1 ng/ml	0.441 (NR)	0.67.6 (NR)	0.35 (0.226–0.474)
van Wolfswinkel_2016	6	0	9	24	ELISA	On admission	1.83 ng/ml	1 (0.54–1.00)	0.52 (0.54–0.87)	0.87 (0.75–0.99)
Torregrosa_2014	14	6	45	79	ELISA	12 h after surgery	1.73 ng/ml	0.716 (NR)	0.64 (NR)	0.713 (0.551–0.876)
Tekce_2014	7	1	1	13	ELISA	On first day after treatment	1.412 ng/ml	0.875 (NR)	0.933 (NR)	0.94 (0.75–0.99)
Nickolas_2012	50	46	323	1216	chemiluminescent microparticle immunoassay	Within 12 h after patient enrollment	2.817 ng/ml	0.52 (NR)	0.79 (NR)	0.71 (0.65–0.76)
Naggar_2012	18	2	1	19	ELISA	Within 1 day after patient enrollment	NR	0.909 (NR)	0.952 (NR)	NR (NR)
Endre_2011	102	154	52	220	microsphere-based Luminex xMAP technology	NR	1.86 ng/mg	0.40 (0.32–0.48)	0.81 (0.77–0.85)	0.66 (0.61–0.72)
Liang_2010	27	3	20	72	ELISA	12 h after surgery	2.0 ng/mg	0.90 (NR)	0.788 (NR)	0.882 (0.812–0.934)
Ferguson_2010	71	21	0	42	ELISA	NR	1.7 ng/mg	0.77 (0.67–0.85)	1 (0.92–1.0)	0.89 (0.82–0.94)
Liangos_2009	12	1	38	52	ELISA	2 h after surgery	0.42 ng/mg	0.92 (NR)	0.58 (NR)	0.78 (0.64–0.91)
Han_2009	19	17	12	42	ELISA	Post-operation Immediately	1.2 ng/mg	0.5143 (NR)	0.7778 (NR)	0.68 (0.58–0.78)
Vaidya_2008	92	10	4	98	ELISA	The time of initial consultation	0.70 ng/mg	0.90 (NR)	0.96 (NR)	0.95 (0.90–0.98)

*KIM-1* kidney injury molecule 1, *AUC* area under curve, *CI* confidence interval, *TP* true positive, *FN* false negative, *FP* false positive, *TN* true negative, *ELISA* enzyme-linked immunosorbent assay, *CA* coronary angiography, *NR* not reported

We also performed subgroup analysis based on different standards, such as study design, population settings, the use of blinding and assay method. The results of the subgroup analysis are shown in Table 4. Nonprospective studies were notably more sensitive and specific than prospective studies. For population settings, patients who underwent CPB showed lower specificity and emergency status, and critical patients showed lower sensitivity than others. Whether blindness was used in the research also had an impact on the results. It was also shown that detection by ELISA was significantly more sensitive than non-ELISA method.

**Table 4:** Subgroup analysis based on different standard

Studies		Sensitivity (95% CrI)	Specificity (95% CrI)	+ LR (95% CrI)	− LR (95% CrI)	DOR (95% CrI)	RD (95% CrI)	AUC (95% CrI)
All studies (14)		0.74 (0.62–0.84)	0.84 (0.76–0.90)	4.58 (2.78–9.47)	0.34 (0.23–0.46)	15.22 (6.74–42.20)	0.55 (0.43–0.70)	0.62 (0.41–0.76)
Study design								
Prospective (9)		0.65 (0.51–0.81)	0.76 (0.70–0.82)	2.75 (2.47–3.21)	0.47 (0.24–0.62)	6.68 (4.00–13.18)	0.41 (0.30–0.56)	0.73 (0.67–0.85)
Non-prospective (5)		0.82 (0.66–0.92)	0.91 (0.74–0.99)	9.36 (2.48–24.05)	0.17 (0.07–0.27)	57.21 (16.26–134.38)	0.67 (0.53–0.78)	0.69 (0.30–0.95)
Blinding or not								
Blinding (5)		0.68 (0.43–0.89)	0.76 (0.68–0.82)	2.97 (2.49–3.53)	0.37 (0.24–0.55)	9.18 (4.62–14.49)	0.48 (0.35–0.58)	0.76 (0.64–0.82)
Non-blinding (9)		0.77 (0.65–0.88)	0.88 (0.78–0.96)	6.29 (3.89–12.03)	0.25 (0.17–0.32)	28.64 (12.26–72.31)	0.65 (0.55–0.77)	0.64 (0.24–0.89)
Population settings								
CPB surgery (4)		0.73 (0.45–0.93)	0.77 (0.62–0.90)	4.06 (2.10–7.53)	0.41 (0.31–0.64)	11.75 (3.50–23.51)	0.47 (0.27–0.61)	0.75 (0.49–0.90)
Emergency and critical patients (4)		0.62 (0.365–0.857)	0.79 (0.66–0.91)	3.79 (1.00–6.96)	0.44 (0.22–0.72)	13.25 (2.82–31.69)	0.45 (0.22–0.68)	0.61 (0.44–0.80)
Others (6)		0.80 (0.66–0.91)	0.89 (0.75–0.97)	7.73 (5.28–13.56)	0.18 (0.16–0.22)	44.44 (24.23–85.74)	0.71 (0.66–0.79)	0.75 (0.54–0.91)
Assay method								
ELISA (12)		0.78 (0.68–0.88)	0.85 (0.75–0.92)	5.59 (3.05–10.18)	0.26 (0.15–0.34)	25.04 (10.33–65.82)	0.63 (0.52–0.77)	0.66 (0.41–0.89)
Non-ELISA (2)		0.45 (0.27–0.65)	0.80 (0.72–0.86)	2.43 (2.35–2.51)	0.65 (0.62–0.67)	3.78 (3.50–4.05)	0.28 (0.26–0.30)	0.55 (0.53–0.57)

*CrI* credible interval

## Discussion

Early diagnose of AKI plays a major role in its treatment and prognosis. At present, AKI is usually diagnosed by increased serum creatinine or reduced urine output. However, current diagnostic criteria cannot meet current clinical needs. There is an urgent need to find a more effective diagnostic measurement for AKI. Some new biomarkers are considered to have the potential to help diagnose AKI early, one of which is urinary KIM-1.

In this diagnostic meta-analysis, we included all published studies that evaluated the diagnostic value of urinary KIM-1. We identified 14 eligible studies, from which we extracted relevant information. We found that urinary KIM-1 can help diagnose AKI with high sensitivity and specificity. Pooled analysis of the studies shows that the estimated sensitivity of urinary KIM-1 in the diagnosis of AKI was 0.74, while the specificity was 0.84. The result is similar to that of the study by Shao et al. [15] in 2014, which proposed the use of urinary KIM-1 in the diagnosis of AKI with a sensitivity of 0.74 and a specificity of 0.86. The main difference between these two studies was the age of the research objects. Our research focus on patients above 18 years old, but the study by Shao et al. [15] included patients of all ages. In addition, the pooled DOR was 15.22(95% CrI, 6.74–42.20), which suggested that uKIM-1 had good diagnostic effectiveness for AKI. When the DOR is greater than 1, higher diagnostic odds ratios are indicative of better test performance [33]. The crosshair plot showed that the sensitivity and specificity of the included studies varied, with some demonstrating rather different values. The results also suggested the need to conduct a meta-analysis to summarize the study findings. The SROC plot combined the sensitivity and specificity of each study and the pooled sensitivity and specificity, which helped emphasize and visualize the difference. The area under the curve was greater than 0.5, which suggested that uKIM-1 had good predictive value for AKI.

To fully evaluate the diagnostic value of uKIM-1, we conducted subgroup analysis based on different standards, such as study design, population setting, the use of blinding and assay method. The results did not show that the factors in the subgroup analysis had a significant influence on the diagnostic value of uKIM-1, except when using non-ELISA methods to measure uKIM-1. Nonprospective studies were notably more sensitive and specific than prospective studies, probably because nonprospective studies enrolled established AKI patients. The non-ELISA methods (chemiluminescent microparticle immunoassay or microsphere-based Luminex xMAP technology) showed much lower sensitivity than ELISA. Although ELISA is the main method of measurement, not all studies used the same kits. The various antibodies and reagents had an impact on test performance, which also made it difficult to analyze the differences in this regard. The time to collect samples and measure uKIM-1 plays an important role in diagnosing AKI [3]. Because the included studies varied in population setting and time of measurement, it was difficult for us to perform a subgroup analysis based on time.

However, KIM-1 is not ready for clinical practice. Our research indicated that there is a relatively large difference in the cutoff value used for KIM-1. The absolute values ranged from 1.412 ng/ml to 3.1 ng/ml, while standardized cutoff values ranged from 0.42 ng/mg to 2.0 ng/mg; given the factors we mentioned before, including population setting, time of measurement, assay method and so on, it is still difficult to determine a suggested cutoff value of KIM-1. In addition, the estimated sensitivity and specificity of urinary KIM-1 in the diagnosis of AKI were 0.74 (95% CrI, 0.62–0.84) and 0.84 (95% CrI, 0.76–0.90), respectively. Neither are sufficiently high for clinical diagnosis. In the present study, we were only able to distinguish whether a patient with a particular KIM-1 value had AKI, but we could not determine the changes in the KIM-1 value as the kidney disease progressed. If the original studies would have included additional laboratory indexes showing kidney disease progression and KIM-1 values in different stages, we would have been able to evaluate the diagnostic value of uKIM-1 more accurately. The KIM-1 value in different stages of kidney disease is meaningful for emergency and critical patients, as many studies suggest that KIM-1 can distinguish patients with diverse types of acute tubular necrosis from those without AKI [34]. To date, no new biomarker has been universally applied in routine use for clinical practice because each biomarker has its own advantages and disadvantages [35]. Thus, an increasing number of researchers have proposed the need for a panel of kidney-specific biomarkers that can reflect functional as well as structural damage and recovery [36]. The combined panel of normalized urinary hemojuvelin and uKIM-1 was reported to have a sensitivity of 1.00 and specificity of 0.70 [37]. A combined panel of kidney-specific biomarkers can provide more directions for biomarker diagnosis and is worthy of deeper exploration in the future research.

## Conclusion

In summary, compared with the current literature, this meta-analysis included updated clinical studies and used more accurate analysis methods to evaluate the diagnostic value of urinary KIM-1 in adults. Our analysis results indicate that uKIM-1 is a good predictor for AKI in adult patients with relatively high sensitivity and specificity. However, further research and clinical trials are still needed to confirm whether and how uKIM-1 can be widely used in clinical diagnosis. In the future, we expect KIM-1 or other kidney biomarkers to be comprehensively applied in AKI, from clinical detection to treatment and even prevention.

## Data Availability

All data analyzed during this study are available in the public domain.

## Abbreviations

AKI: Acute kidney injury
AKIN: Acute kidney injury network
AUC: Area under curve
CA: Coronary angiography
CI: Confidence interval
CrI: Credible interval
CPB: Cardiopulmonary bypass
DIC: Deviance information criterion
DOR: Diagnostic odds ratio
eGRF: Estimated glomerular filtration rate
ELISA: Enzyme-linked immune sorbent assay
FDA: Food and Drug Administration
FN: False negative
FP: False positive
ICU: Intensive care unit
IL-18: Interleukin-18
KDIGO: Kidney Disease Improving Global Guidelines
KIM-1: Kidney injury molecule 1
NGAL: Neutrophil gelatinase-associated lipocalin
PRISMA: Preferred reporting items for systematic reviews and meta-analyses
QUADAS-2: Quality assessment tool for diagnosis accuracy studies
RD: Risk difference
RIFLE: Risk, injury, failure, loss, end stage kidney disease
SCr: Serum creatinine
SROC: Summary receiver operating characteristic
TIMP-2: Tissue inhibitor of metalloproteinase 2
TN: True negative
TP: True positive
uKIM-1: Urinary kidney injury molecule 1
